# The Lyell Collection at the Earth Sciences Department, Natural History Museum, London (UK)

**DOI:** 10.3897/BDJ.7.e33504

**Published:** 2019-02-19

**Authors:** Consuelo Sendino

**Affiliations:** 1 Natural History Museum, London, United Kingdom Natural History Museum London United Kingdom

**Keywords:** Sir Charles Lyell, Macaronesia, Canaries, Madeira, Cenozoic, digitisation, collection.

## Abstract

**Background:**

This paper provides a quantitative and general description of the Lyell Collection kept in the Department of Earth Sciences at the Natural History Museum of London. This collection started to be built by the eminent British geologist Sir Charles Lyell (1797-1875) in 1846 when the first specimen reached the Museum. The last one entered in 1980 donated by one of Lyell’s heirs. There are more than 1700 specimens, mainly hand specimens with 93% of the fauna and flora from the Cenozoic of the Macaronesian archipelagos of the Canaries and Madeira. Those specimens that belong to the Lyell Collection with certainty have been databased and imaged. Currently they are being geo-referred automatically with the rest of the site geo-references at the NHM. This collection could be increased by a couple of dozen more specimens with those specimens located in the same drawers, but they do not have collector details. The work of data collection of these specimens was implemented over a year from 2016 to 2017, including annelids; brachiopods; bryozoans; echinoderms; scyphozoans; bivalves; gastropods; scaphopods; trilobites; plants; reptiles; fishes; and mammals.

**New information:**

Access to the specimen-level data is available through the NHM data portal with the images associated. This is the first time that a description of the Fossil Lyell Collection dataset is available in the literature.

## Introduction

Sir Charles Lyell, 1^st^ Baronet, (14 November 1797 – 22 February 1875) was a lawyer and geologist whose work influenced both Darwin and Wallace and has been accepted as one of the greatest geological thinkers of the 19^th^ century. His work will be remembered among many other achievements for devising theories for earthquakes, volcanoes and stratigraphy and coining the terms for the Paleozoic, Mesozoic and Cenozoic geological eras. His main work is displayed in his Principles of Geology (1830-1833) (Lyell 1830, Lyell 1832, Lyell 1833 and made him regarded as the pioneer of modern geology.

The Fossil Lyell Collection started to be assembled in June 1846 when C. Lyell Esq., as it is recorded in the NHM catalogue books, presented *Cephalaspis lyelli* from the Old Red Sandstone of Glammis, Forfarshire (Scotland), figured by Agassiz in 1835 [NHMUK PV OR 20087] (Agassiz 1835). From then until the last specimen that entered the NHM in 1980, presented by Lady Sophie Mary Lyell (1916 - 2012), a shark tooth, *Carcharocles angustidens* (Agassiz, 1843), 1,735 specimens have been recorded. There are 13 additional specimens that were probably collected by Charles Lyell, although these ones do not have any label with them; some have numbers glued with similar handwriting of that of Charles Lyell.

This collection is mainly composed of fossil specimens, but there are also 51 recent brachiopods, including the 9 collected by Charles Darwin (1809-1882) from Tierra del Fuego or Galapagos during his trip on board the Beagle and later given to Charles Lyell. They are mostly isolated hand specimens with the exception of 7 bryozoan cavity slides and 73 mounted bryozoans (with Lyell’s original handwriting) and 49 mounted brachiopods. There are in total 51 type and figured specimens distributed among molluscs (25), bryozoans (24), fish (1) and reptiles (1), in decreasing number (see Table 1). Finally, there 35 cited specimens: 33 brachiopods and one coelenterate cited in one of the Lyell’s publications (Lyell 1845) and the other cited specimen is a bivalve mentioned by Murchison (Murchison 1829).

The importance of this collection is not only historical, but also scientifically significant as being the main reason of the stratigraphic and volcanic studies that Lyell carried out in the Canaries, Madeira and Sicily. These specimens are fundamental to understand Lyell’s theory on volcano formation and the 19th century theory on uniformitarianism. Most of them come from sites that currently are resorts, sites where collecting is not possible. Therefore they enrich Natural History Museum collections and are important for Science and British National Heritage.

The size of this Collection makes it ideal to test a pilot project on digitisation of specimens scattered in the collections. A pre-study of the catalogue books made us think that there were about 700 specimens, but after the search there were more than 1,700 specimens.

## General description

### Purpose

**Figure 1.**
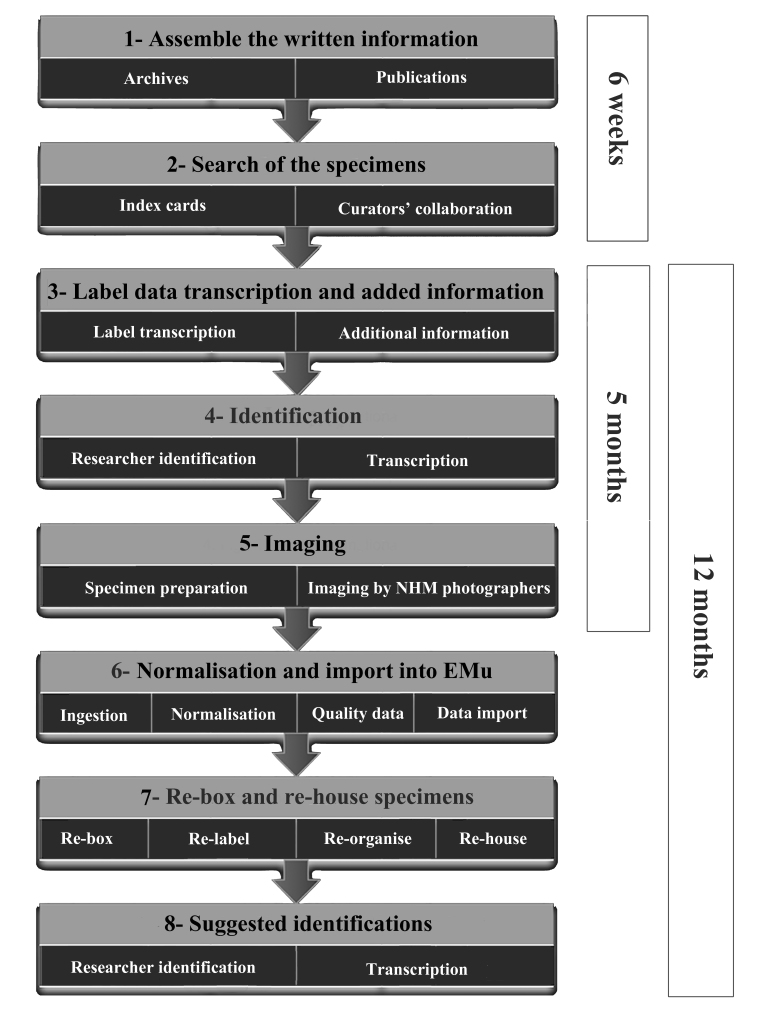
Project workflow.

To digitise this collection, as part of the Museum Strategy, a pilot project was created with the different steps to achieve the whole digitization of the collection (Fig. 1). This included photographs of the specimens that currently are available through the NHM Data Portal.

### Additional information

**Collection digitisation history**

To digitise this collection, as part of the Museum Strategy, a pilot project was created with the different steps to achieve the whole digitization of the collection. This included photographs of the specimens that currently are available through the NHM Data Portal.

The beginning of the project (Fig. 1) involved assembling all the written information recorded in the NHM during those dates compatible with Lyell’s life and those of his direct heirs (from 1840 to 1915). The reason for the latter date (1915), after Lyell’s death, is because Charles Lyell’s nephew, Sir Leonard Lyell, donated some of Charles Lyell’s specimens in 1913, as recorded in the *World Palaeontological Collections* by Cleevely (Cleevely 1983); then a few later years were added to the search just in case there was another donation not documented by Cleevely. We avoided searching up to the last donation in 1980 to narrow the search time. At this stage most of the work was done in the Library, mainly searching in the old catalogue books and archives where original letters from Charles Lyell are preserved. We also studied other publications from during Lyell’s lifetime where his collection specimens were described and/or cited (see bibliographical references).

**Figure 2.**
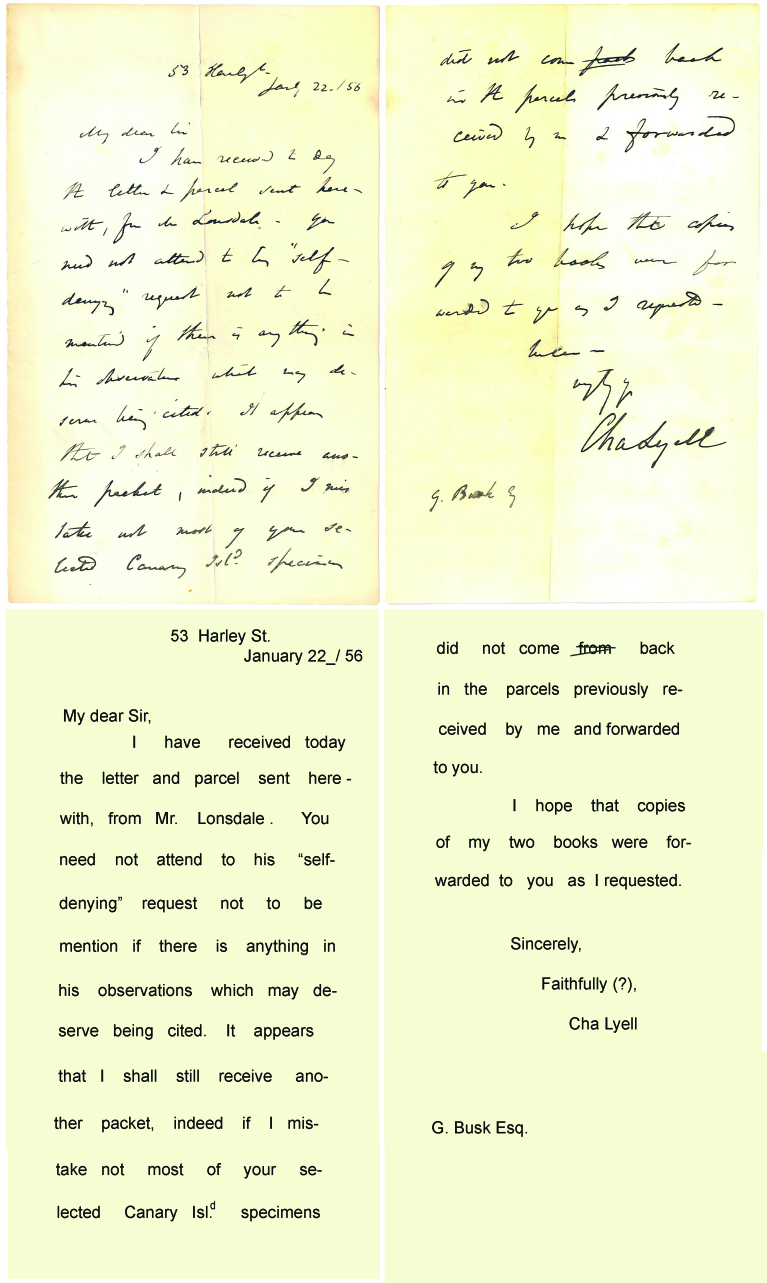
Letter sent by Lyell to Busk on 22 January 1856 on identification of the bryozoans from the Canaries with a transcription below.

The next step was the search of the specimens. According to the register books, we were able to restrict the search to specific group collections. At this stage the use of the index cards and the collaboration of colleagues were really valuable. The data collected from the specimens’ labels and letters associated to the specimens (Fig. 2) including verbatim data: species name; geographical details; stratigraphy; and collection details (collector, collection and date if available), was digitised, following a transcription protocol. At the same time that the data was recorded, the specimens were identified with help of an NHM scientific associate (retired researcher). After this, the specimens were mostly photographed by the Museum photographers and an NHM visitor researcher. The 1030 images were stored on TIFF (998) and JPEG (32) files respectively on the server waiting for the remaining data to be imported into Emu, an estimated total of 78 GB from 15 batches. Some of these images were rotated to show the conventional position for those which did not have the right orientation and cropped with Adobe Photoshop CC 2014. The image names given by the photographers were adopted. They are based on the registration name and the shot number. The Museum photographers used the focus stacking technique as most of the specimens are preserved in three dimensions and it is needed for keeping the finest detail.

Once all the specimen data -of the Library and specimen labels- was recorded on an Excel spreadsheet, an appropriate template for the information collected, it was processed (ingestion, normalisation with the coinciding data in Emu -master records- and quality data) and uploaded into EMu (Sendino 2009), the NHM’s collection management system. Data normalisation is basic to avoid data duplication, conflict with pre-existing data and to remove and avoid erroneous records. New taxonomic, stratigraphic and geographic Emu records were only recorded if not already present. The reason to use Excel instead of Emu directly is because Excel allows rapid data entry with copying tools that facilitate this process. The quality control and assurance procedures are implemented at all stages as errors can occur at any stage of the digitisation, but quality control is precisely required on the workflow modules 3 and 6. Currently, this data is available through the NHM Data Portal. Finally, all the specimens have been re-boxed and re-labelled and mostly (molluscs and annelids) re-housed to a new location to facilitate their easy access for research and future tours behind the scenes; also new identifications have been suggested in determination comments. These are not definitive as the specimens are in need of further research.

The first two stages of this project, collecting all the information about the specimens and locating them in the collections, were carried out over a six week period thanks to cooperation with the University of Valencia (Spain) which funded an MSc student (Sáez Máñez 2017). These modules of the workflow are independent and were performed sequentially. The rest of the project was carried out over approximately 24 months part time (or 12 months full time) and whose modules (from 3 to 8) were executed in parallel (Fig. 1). For instance, part of the transcription was done at the same time as the specimens were prepared for being imaged and re-boxed and re-labelled. The re-boxing was done with the help of a single volunteer who re-boxed more than 1,500 specimens. The new labels were done with special paper and pens for archival purposes according to the NHM conservation standards, including secol to cover the new labels and those pieces of papers with Lyell’s original handwriting and former identifications. They also include DataMatrix barcodes for easier access to the specimens data through the NHM collection management system.

The last stage of the project was the update of the taxonomic identifications where possible, giving the suggested identifications in the determination comments. Lyell’s identifications –assuming those recorded in the catalogue books–, researcher’s identifications – as those given by the Spanish researcher Joaquin Meco in 1973; M. B. Sedda in 1992 and Noel Morris in 2017 & 2018- and suggested taxonomic names are displayed through the NHM Data Portal with the dates (approximate dates for Lyell’s) for these specimens.

The digitisation workflow has been an important way of recording decisions, above all the first stages that addressed the following steps. The most consuming time modules were the data label transcriptions, data normalisation and suggested taxonomic identifications.

**Historical Collection**

**Figure 3.**
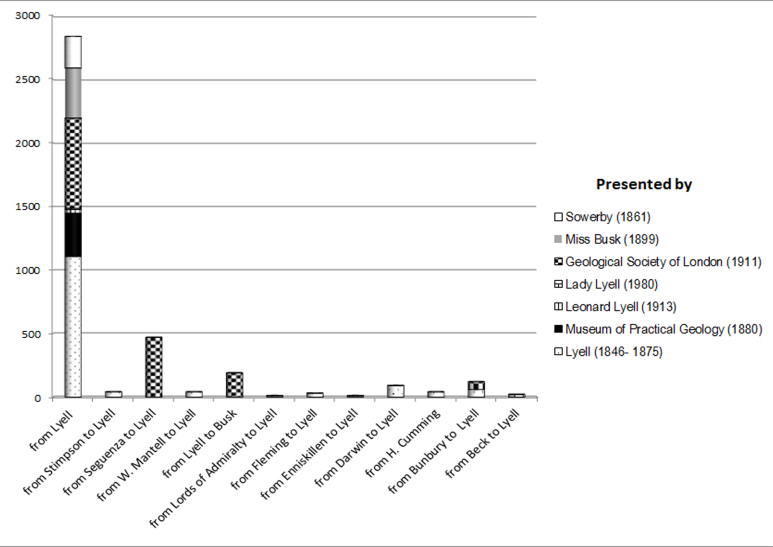
Origin of the Lyell Collection. The scale of those specimens which are fewer than 100 has been increased 10 times to enhance their visibility. Please see Suppl. material 1 for further information.

The origin of the different collections integrated into the Lyell collection had different sources, such as Lyell’s specimens from the Geological Society that entered in the Museum in 1911 (almost 5%, 1 is from Miss Busk, George Busk’s daughter) or those that came from Museum of Practical Geology in 1880 (almost 2%) and directly from Miss Busk in 1899 (almost 23%) and James Sowerby (0.4%) in 1861. Lyell was also the receiver of other people’s specimens [Charles Darwin’s brachiopods -0,5%-; Charles James Fox Bunbury’s gastropods -0.6%-; Giuseppe Seguenza’s molluscs from Sicily –almost 3%-; Dr Beck’s brachiopods -0.1% -; H. Cumming’s brachiopods -0.1%-; Dr Fleming’s brachiopods -0.1%-; William Mantell’s -0.2%- brachiopods; Lords of Admiralty’s and W. Stimpson’s brachiopods from the Gulf Stream Expedition -0.2%-; and William Willoughby Cole Enniskillen’s (3rd Earl) fish –only one specimen-]. But the main part of the collection came from 1846 to 1875, the latter is the year of his death, with more than 52% of the specimens. As already noted above, there have been fossils that were presented by Lyell’s successors: Leonard Lyell donated three fishes in 1913 and Lady Lyell one fish in 1980). 13% of them do not have the date when Lyell presented them to the NHM (Fig. 3).

This collection is not only key on research of volcano formation and palaeontology of Macaronesia (93% of the palaeoflora and palaeofauna are from the North Atlantic Ocean), but it is also reference for scientific and taxonomic studies (1.5% contains types).

## Geographic coverage

### Description

**Figure 4.**
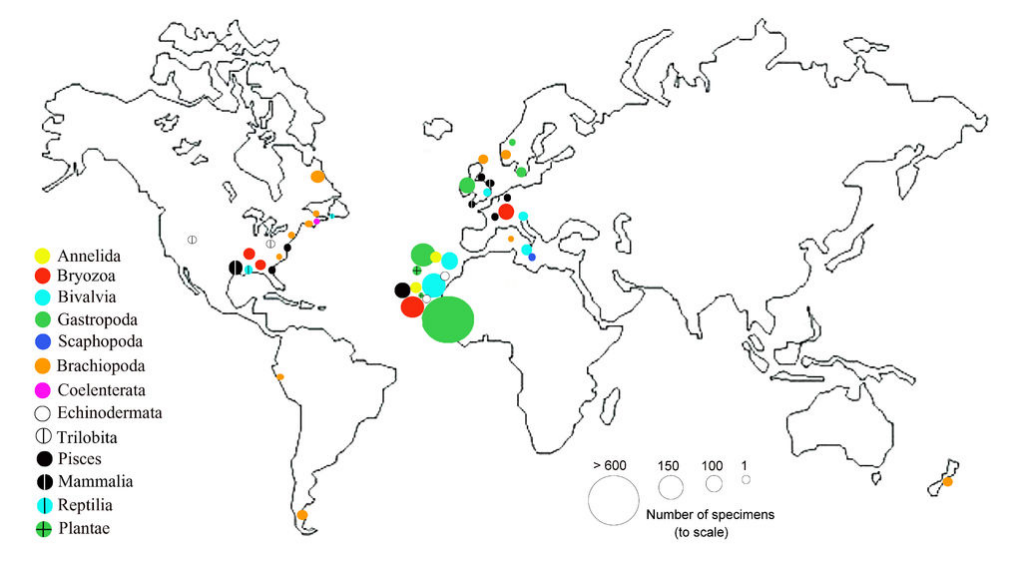
Bubble map with the sites from where the Earth Sciences’ Lyell Collection specimens originate.

**Figure 5.**
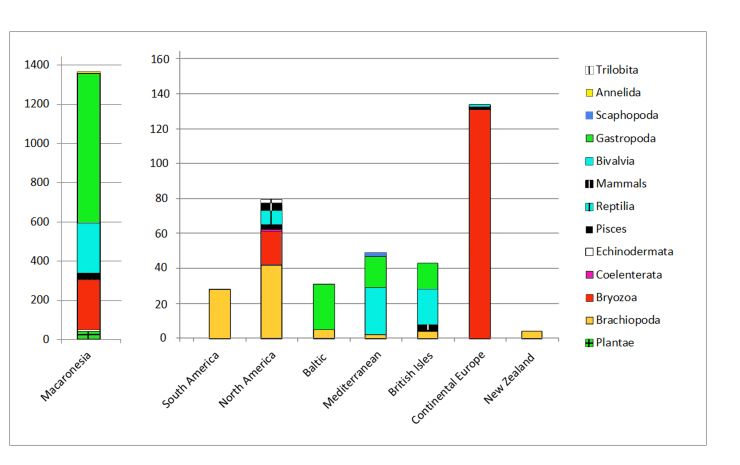
Number of specimens per region and group; note the two different scales.

At this stage of the NHM digitisation development, most of the specimens have been assigned geographic coordinates (Fig. 4) automatically with the rest of the site geo-references at the NHM. The georeferencing tool used allows a user to acquire point and extent data from Google Maps, following the NHM Georeferencing standards based on best practice and is freely available at the NHM. Most of the Lyell Collection, 93%, comes from Macaronesia (Fig. 5).

### Coordinates

 and Latitude; and Longitude.

## Taxonomic coverage

### Description

This collection covers two kingdoms, Plantae (2.26% of the sample) and Animalia (97.74%).

**Figure 6.**
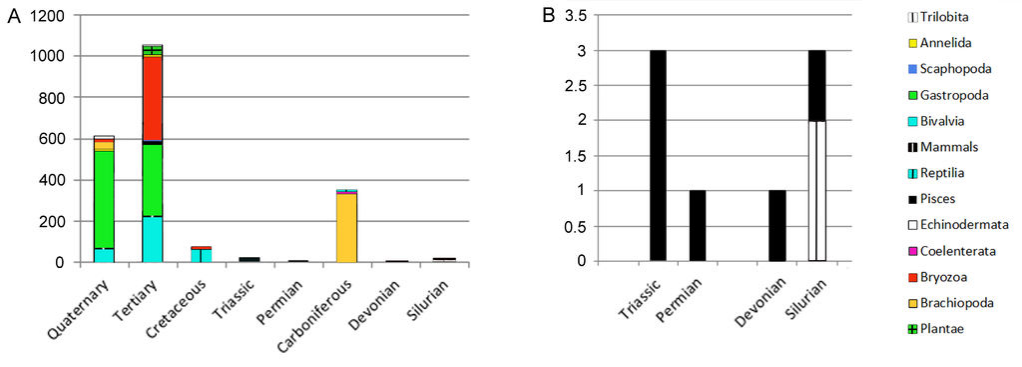
6A. Number of specimens per stratigraphic period and group. 6B. For further clarification for Silurian, Devonian, Permian and Triassic.

Plantae includes the groups Chlorophyta (0.11% of the sample and 5% of plants); Pteridophyta (0.5% of the sample and 23 % of plants); Embryophyta (0.58 % of the sample and 25 % of plants); and Magnoliophyta (1% of the sample and 46 % of plants). See Figs 5, 6 for stratigraphic and taxonomic coverage of plants.

Animalia includes 8 phyla: Annelida (0.3% of the sample and 0.3% of animals); Bryozoa (24% of the sample and 24.5% of animals); Mollusca (64.21% of the sample and 66% of animals); Brachiopoda (4.9% of the sample and 5% of animals); Coelenterata (0.05% of the sample and 0.05% of animals); Echinodermata (0.46% of the sample and 0.47% of animals); Trilobita (0.11% of the sample and 0.11% of animals); and Chordata (2.49% of the sample and 2.55% of animals). See Figs 5, 6 for stratigraphic and taxonomic coverage of animals.

Most of the molluscs had no identification and the others had obsolete taxonomic names. All the specimens have been identified as much as their preservation allowed and through the knowledge of the NHM research associates. The obsolete names have been suggested to be changed to the updated ones on the NHM Data Portal.

## Temporal coverage

### Notes

As a palaeontological collection, its stratigraphical distribution is important. They are mainly Cenozoic specimens, largely from the Tertiary (more than 61%) and Quaternary (more than 35%) of Macaronesia. The rest of the specimens are from the Silurian to Cretaceous and are in very low numbers (Fig. 6). This collection was mostly made to help in understanding volcano formation.

Lyell visited the Canaries and the Madeira islands from December 1853 to April 1854. He was guided around Gran Canaria by the Spanish engineer Pedro Maffiotte (1816-1870) and around Madeira by the Portuguese military engineer Major Antonio Azevedo and the German Georg Hartung (1821–1891), who had an interest in geology. All of them contributed to Lyell’s collection. Maffiotte posted to Lyell further material from the localities that they had visited together and exchanged letters with him for twelve years between 1854 and 1866. Lyell spent several years studying the specimens collected from these islands, and exchanged letters with other researchers in an attempt to determine the identities of the species present.

## Collection data

### Collection name

Lyell Collection

### Specimen preservation method

Isolated and mounted dried specimens

### Curatorial unit

Palaeobiological collections

## Usage rights

### Use license

Open Data Commons Attribution License

## Data resources

### Data package title

The Charles Lyell fossil collection at the Natural History Museum

### Resource link

http://data.nhm.ac.uk/dataset/the-charles-lyell-fossil-collection-at-the-natural-history-museum

### Number of data sets

3

### Data set 1.

#### Data set name

The Charles Lyell fossil collection at the Natural History Museum

#### Data format

TIFF

#### Number of columns

1

#### Download URL

http://data.nhm.ac.uk/dataset/the-charles-lyell-fossil-collection-at-the-natural-history-museum

#### Description

Photographs of the specimens and an article on the planification of the Lyell Project

**Data set 1.** 

Column label	Column description
Project	Narrative

### Data set 2.

#### Data set name

Lyell Collection-Types and figured and cited specimens

#### Data format

Word

#### Number of columns

5

#### Download URL

http://data.nhm.ac.uk/dataset/lyell-collection-types

#### Description

Types and cited specimens included in the Fossil Lyell Collection (group; type status; identification; and reference).

**Data set 2.** 

Column label	Column description
Phyllum	Phylum names
Type/Cited	Types and figured and cited specimens' names
Specimen number	Registration number
Taxon	Published taxon
Reference	Publication

### Data set 3.

#### Data set name

Lyell Collection-List of specimens

#### Data format

CSV

#### Number of columns

4

#### Download URL

http://data.nhm.ac.uk/dataset/list-specimnes-lyell-collection

#### Description

Lyell Collection with identifications and specimen numbers

**Data set 3.** 

Column label	Column description
Registration-No.:(Registration)	Specimen number
Description:(Catalogue)	Group
Taxon:(Determination-Details)/Taxonomy:(Object-Details)	Identfication
Number:(Type-And-Number-Of-Objects)	Number of specimens

## Additional information

**Reduced project cost**

This pilot project has been carried out with few resources. Regarding staff working on this, there was a curator involved in the whole project; an MSc student working only 6 weeks on the project; a volunteer working a day per week over a year; an NHM photographer working for 3 months full time; a research associate working a day per week over two months giving broad IDs; and less than one day for each curator with Lyell specimens in their collections. Other additional costs comprise consumables such as plastazote, acid free trays, archival pens, and archival paper for new labels. The success of this was due to advanced planning and resource tracking.

Each stage accomplishes a particular objective and creates inputs to the next stage (Fig. 1). The framework may be implemented in the form of a decision support system, and a prototype system is described which supports many of the related decision making activities.

This is a good example of reduced cost for digitisation infrastructure creation maintaining a high public profile for digitisation.

**Collection results**

A scattered and unrecorded collection has been assembled, recorded, photographed and displayed through the NHM Data Portal that currently is under the Beta version. The taxonomic names given by Lyell, where these have been preserved, are respected, but also suggested taxonomic names are displayed in comments through the NHM Data Portal. The importance of this project lies in the Collection being ready for research, not only on taxonomy, but also for stratigraphic and volcanic formation studies, as 93% of the specimens come from the Macaronesia islands.

The specimens have been re-housed in plastazote, acid free trays in a new location with all the molluscs in the same cabinet, with easier access for research and for salvage purposes.

The display of this data virtually eliminates the need for specimen handling by researchers and will greatly speed up response time to collection enquires.

This pilot project procedure and its workflow are advantageous for new digitisation projects regarding collections containing a variety of specimens of different taxonomic groups.

## Supplementary Material

Supplementary material 1 Appendix Data type: Origin of the Lyell Collection
Brief description: The origin of the different collections integrated into the Lyell collection by different sources
File: oo_262968.csv Consuelo Sendino

## Figures and Tables

**Table 1.** Types and cited specimens included in the Fossil Lyell Collection (group; type status; identification; and reference).

**Phylum**	**Type / Cited**	**Specimen number**	**Taxon**	**Reference**
Bryozoa	Holotype	NHMUK PI D 53190	*Hippothoa tuberculum*	Lonsdale 1845a: p.527.
		NHMUK PI D 53191	*Eschara viminea*	Lonsdale 1845a: p. 531.
		NHMUK PI D 53194	*Eschara linea*	Lonsdale 1845a: p. 530.
		NHMUK PI D 53195	*Cellepora umbilicata*	Lonsdale 1845a: p. 507.
		NHMUK PI D 53198	*Cellepora similis*	Lonsdale 1845b: p. 509.
		NHMUK PI D 53204	*Eschara tubulata*	Lonsdale 1845a: p. 528.
		NHMUK PI D 53205	*Eschara petiolus*	Lonsdale 1845a: p. 529.
		NHMUK PI D 53206	*Eschara incumbes*	Lonsdale 1845a: p. 529.
	Syntype	NHMUK PI D 53201 [3 specimens]	*Escharina tumidula*	p. 502.
		NHMUK PI D 53192	*Cellepora quadrangularis*	Lonsdale 1845b: p. 508.
		NHMUK PI D 53193	*Cellepora quadrangularis*	Lonsdale 1845b: p. 508.
		NHMUK PI D 53196	*Cellepora informata*	Lonsdale 1845b: p. 506.
		NHMUK PI D 53197	*Cellepora informata*	Lonsdale 1845b: p. 505.
		NHMUK PI D 53200	*Cellepora quadrangularis*	Lonsdale 1845b: p. 508.
		NHMUK PI D 53207	*Lunulites contigua*	Lonsdale 1845a: p. 533, Fig. C.
		NHMUK PI D 53208	*Lunulites contigua*	Lonsdale 1845a: p. 533, Fig. a+b.
		NHMUK PI D 53209	*Lunulites distans*	Lonsdale 1845a: p. 531. Figs b+d.
		NHMUK PI D 53210	*Lunulites distans*	Lonsdale 1845a: p. 531.
	Figured	NHMUK PI D 6523	* Labioporella *	Sendino and Taylor 2013: p. 234, Fig. 3G
		NHMUK PI D 6527(a)	* Cryptosula *	Sendino and Taylor 2013: p. 234, Fig. 3F
		NHMUK PI D 6527(b)	* Onychocella *	Sendino and Taylor 2013: p. 234, Fig. 3G
		NHMUK PI D 6535	* Trypostega *	Sendino and Taylor 2013: pp 236-7, Fig. 4C
Mollusca	Holotype	NHMUK PI OR 43119	*Venericardia deltoidea*	Sowerby 1821: p. 106, pl. 259: Fig. 1
		NHMUK PI OR 43540	*Lymnaea columellaris*	Sowerby and Sowerby 1829: p. 53, Pl. 528: Fig. 2
	Syntype	NHMUK PI LL 17678	*Unio solandri*	Sowerby and Sowerby 1829: p. 30. Pl. 517: Upper figure
		NHMUK PI LL 17679	*Unio solandri*	Sowerby and Sowerby 1829: p. 30. Pl. 517: Probably bottom left figure
		NHMUK PI OR 43039(1)	*Crassatella plicata*	Sowerby 1823: p. 62, pl. 545: Fig. 2 left
		NHMUK PI OR 43039(2)	*Crassatella plicata*	Sowerby 1823: p. 62, pl. 545: Fig. 2 centre
		NHMUK PI OR 43039(3)	*Crassatella plicata*	Sowerby 1823: p. 62
		NHMUK PI OR 43039(4)	*Crassatella plicata*	Sowerby 1823: p. 62
		NHMUK PI OR 43548 (1)	*Melanopsis carinata*	Sowerby and Sowerby 1829: p. 41, Pl. 523: Fig. 1, left
		NHMUK PI OR 43776	*Cancellaria laeviuscula*	Sowerby 1823: p. 84, pl. 361: Fig. 1
		NHMUK PI OR 43798 (1)	*Voluta geminata*	Sowerby 1823: p. 136, pl. 398: Fig. 1
		NHMUK PI OR 43798 (2)	*Voluta geminata*	Sowerby 1823: p. 136
		NHMUK PI OR 43798 (3)	*Voluta geminata*	Sowerby 1823: p. 136
	Figured	NHMUK PI OR 44122(1)	*Ostrea flabellula*	Sowerby 1821: p. 97; Pl. 253: figs 2&3;
		NHMUK PI OR 44122(2)	*Ostrea flabellula*	Sowerby 1821: p. 97; Pl. 253: figs 4&6;
		NHMUK PI OR 44122(3)	*Ostrea flabellula*	Sowerby 1821: p. 97; Pl. 253: figs 5
		NHMUK PI OR 44122(4)	*Ostrea flabellula*	Sowerby 1821: p. 97; Pl. 253: figs 7&9
		NHMUK PI OR 43816	*Ancilla subulata*	Sowerby 1823: p. 38, pl. 333: Fig. 4
	Syntype	NHMUK PI OR 43249(1)	*Mytilus Brardii*	Sowerby and Sowerby 1829: p. 61, pl. 532: Fig. 2 (centre of 3).
		NHMUK PI OR 43249(2)	*Mytilus Brardii*	Sowerby and Sowerby 1829: p. 61, pl. 532: Fig. 2 (right of 3).
		NHMUK PI OR 43249(3)	*Mytilus Brardii*	Sowerby and Sowerby 1829: p. 61, pl. 532: Fig. 2 (left of 3).
		NHMUK PI OR 43249(4)	*Mytilus Brardii*	Sowerby and Sowerby 1829: p. 61
		NHMUK PI OR 43249(5)	*Mytilus Brardii*	Sowerby and Sowerby 1829: p. 61
		NHMUK PI OR 43535	*Ancylus elegans*	Sowerby and Sowerby 1829: p. 64; Pl. 533; probably the lower specimen
	Cited	NHMUK PI TB 14570	*Pecten gemellari*	Murchison 1829: p. 403
Coelenterata	Cited	NHMUK PI PG 4320 (=NHMUK PI CL 800)	* Conularia *	Lyell 1845: p. 191
Brachiopoda	Cited	NHMUK PI B 16591	*Spirifer glaber*	Lyell 1845: p. 221, n.36
	Cited	NHMUK PI B 16594	*Producta Martini*	Lyell 1845: p. 221, n. 40
	Cited	NHMUK PI B 16595	*Producta Martini*	Lyell 1845: p. 221, n. 40
	Cited	NHMUK PI B 16601	*Producta Martini*	Lyell 1845: p. 221, n. 40
	Cited	NHMUK PI B 16602	*Producta Scotica*	Lyell 1845: p. 222, n. 43
	Cited	NHMUK PI B 16603	*Producta Martini*	Lyell 1845: p. 221, n. 40
	Cited	NHMUK PI B 16605	*Producta Scotica*	Lyell 1845: p. 222, n. 43
	Cited	NHMUK PI B 16606 [20 specimens]	*Producta Martini*	Lyell 1845: p. 221, n. 40
	Cited	NHMUK PI B 16611	*Producta Martini*	Lyell 1845: p. 221, n. 40
	Cited	NHMUK PI B 16613	*Spirifer glaber*	Lyell 1845: p. 221, n. 36
	Cited	NHMUK PI B 16619	*ProductaSscotica*	Lyell 1845: p. 222, n. 43
	Cited	NHMUK PI B 16626	*Producta Scotica*	Lyell 1845: p. 222, n. 43
	Cited	NHMUK PI B 16629	*Producta Scotica*	Lyell 1845: p. 222, n. 43
	Cited	NHMUK PI B 16633	*Producta Martini*	Lyell 1845: p. 221, n. 40
Chordata	Syntype / Lectotype	NHMUK PV OR 20087	*Cephalaspis lyelli*	Agassiz 1835: pl. 1a, fig. 2. / White 1958: pl. 18, figs 1-2
	Holotype	NHMUK PV R 4168	*Hylonomus lyelli*	Dawson 1860: pp 274-5, figs 14-18.
